# Pro-Apoptotic Activity of the Marine Sponge *Dactylospongia elegans* Metabolites Pelorol and 5-*epi*-Ilimaquinone on Human 501Mel Melanoma Cells

**DOI:** 10.3390/md20070427

**Published:** 2022-06-28

**Authors:** Sara Carpi, Egeria Scoditti, Beatrice Polini, Simone Brogi, Vincenzo Calderone, Peter Proksch, Sherif S. Ebada, Paola Nieri

**Affiliations:** 1 NEST, Istituto Nanoscienze-CNR and Scuola Normale Superiore, 56126 Pisa, Italy; 2 Department of Pharmacy, University of Pisa, 56126 Pisa, Italy; beatrice.polini@farm.unipi.it (B.P.); simone.brogi@unipi.it (S.B.); vincenzo.calderone@unipi.it (V.C.); paola.nieri@unipi.it (P.N.); 3 National Research Council (CNR), Institute of Clinical Physiology (IFC), 73100 Lecce, Italy; egeria.scoditti@ifc.cnr.it; 4 Department of Pathology, University of Pisa, Via Savi 10, 56126 Pisa, Italy; 5 Interdepartmental Center of Marine Pharmacology (*MArinePHARMA*), University of Pisa, 56126 Pisa, Italy; 6 Institute of Pharmaceutical Biology and Biotechnology, Faculty of Mathematics and Natural Sciences, Heinrich Heine University Düsseldorf, Universtätsstrasse 1, 40225 Düsseldorf, Germany; peter.proksch@hhu.de; 7 Department of Pharmacognosy, Faculty of Pharmacy, Ain Shams University, Cairo 11566, Egypt; sherif_elsayed@pharma.asu.edu.eg

**Keywords:** melanoma, pelorol, 5-*epi*-ilimaquinone, marine sponge, apoptosis, microRNA, *Dactylospongia elegans*

## Abstract

The natural environment represents an important source of drugs that originates from the terrestrial and, in minority, marine organisms. Indeed, the marine environment represents a largely untapped source in the process of drug discovery. Among all marine organisms, sponges with algae represent the richest source of compounds showing anticancer activity. In this study, the two secondary metabolites pelorol (PEL) and 5-*epi*-ilimaquinone (EPI), purified from *Dactylospongia elegans* were investigated for their anti-melanoma activity. PEL and EPI induced cell growth repression of 501Mel melanoma cells in a concentration- and time-dependent manner. A cell cycle block in the G1 phase by PEL and EPI was also observed. Furthermore, PEL and EPI induced significant accumulation of DNA histone fragments in the cytoplasmic fraction, indicating a pro-apoptotic effect of both compounds. At the molecular level, PEL and EPI induced apoptosis through the increase in pro-apoptotic BAX expression, confirmed by the decrease in its silencing miR-214-3p and the decrease in the anti-apoptotic BCL-2, MCL1, and BIRC-5 mRNA expression, attested by the increase in their silencing miRNAs, i.e., miR-193a-3p and miR-16-5p. In conclusion, our data indicate that PEL and EPI exert cytotoxicity activity against 501Mel melanoma cells promoting apoptotic signaling and inducing changes in miRNA expression and their downstream effectors. For these reasons could represent promising lead compounds in the anti-melanoma drug research.

## 1. Introduction

The natural environment continues to be an important source of numerous molecular leads for new pharmaceuticals and healthful products [1,2]. Indeed, a large part of marketed therapeutics (about 40%) is based on unmodified or semi-synthetic biologically active natural compounds [3]. In this regard, the majority of drugs derived from natural compounds originate from terrestrial organisms such as plants, microorganisms, and fungi, while marine-derived drugs represent a minority [4]. Marine organisms harbor a great variety of chemical structures in their secondary metabolites, strictly connected to the necessity to survive in hostile habitats. Therefore, the marine environment represents a largely untapped source in the process of drug discovery. In this context, despite significant advances in preventive and therapeutic strategies, cancer remains the second cause of disease-related death in developed countries [5], and there is an urgent need for new anticancer drugs with novel modes of action and fewer side effects. Currently, marine-derived compounds are assuming an increasing role in the search for novel anticancer drug candidates [3,4].

Among all marine organisms, sponges with algae represent the richest source of compounds showing anticancer activity [6,7].

Four molecules derived from marine sponges (or by symbiont cyanobacteria) have already been approved as anti-tumor drugs, cytarabine, fludarabine phosphate, nelarabine, and eribulin mesylate [8]. Cytarabine, fludarabine, and nelarabine are synthetic analogs of spongotimidine or spongouridine, extracted from the *Cryptotethya crypta* sponge. Cytarabine is the first Food and Drug Administration (FDA)-approved marine-derived drug in 1969, while fludarabine phosphate and nelarabine were approved later by the FDA and the European Medicines Agency (EMA). All these drugs act as antimetabolites and are approved for leukemia and lymphoma cancers. Another sponge, *Halichondria okadai*, gave the chemical precursor of eribulin mesylate, approved by the FDA in 2010 and EMA in 2011 for the treatment of metastatic breast cancer [4].

Cutaneous melanoma is one of the most aggressive and fatal forms of skin malignancy, with increasing incidence over the past several decades [9]. Immunotherapy and targeted therapy have significantly improved the outcome of patients with advanced melanoma, but an incomplete therapeutic response, toxic effects, and acquired resistance may limit their use [10,11]. Therefore, melanoma research still needs to develop other treatments that can prolong survival. Marine-derived molecules represent a novel interesting opportunity also for this type of cancer, as documented by in vitro and in vivo evidence [12,13,14].

**Figure 1:**
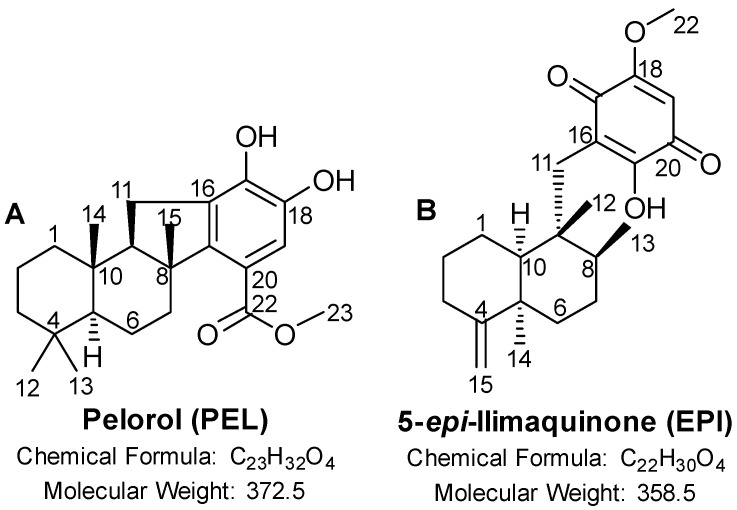
Chemical structure of (**A**) pelorol (PEL) and (**B**) 5-*epi*-ilimaquinone (EPI).

In this study, the two sponge-derived molecules, pelorol (PEL) and 5-*epi*-ilimaquinone (EPI), were investigated for their anti-melanoma activity in order to explore their possible role as new lead anti-melanoma agents. PEL is a meroterpenoid (Figure 1A) previously isolated from the two sponges *Petrosaspongia metachromia* [15] and *Dactylospongia elegans* [16]. PEL showed a wide spectrum of biological activities, such as anti-inflammatory [17,18,19], anti-tumor [20,21], antimicrobial [21], anti-protozoal [16], and antimalarial [16,22] activities. EPI is a sesquiterpene quinone compound (Figure 1B) isolated both from deep and shallow water sponges, including *Dactylospongia elegans* [21,23,24,25], and boasts anti-tumor [21,26] and antimicrobial [21] properties.

In this study, the anti-melanoma activity of PEL and EPI was evaluated for the first time in a cell model of cutaneous melanoma (501Mel), investigating their effects on cell growth and related mechanism(s) of action using functional and transcriptional approaches.

## 2. Results

### 2.1. PEL and EPI Inhibited Melanoma Cell Viability

**Figure 2:**
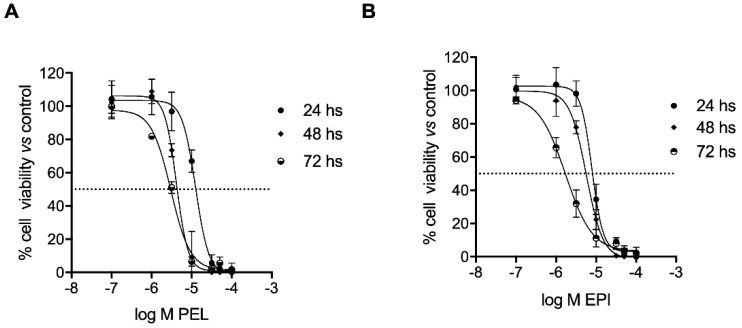
**Pelorol (PEL) and 5–*epi*–ilimaquinone (EPI) reduced the viability of human melanoma cells (501Mel)**. Melanoma cells were treated with increasing concentrations (0.1–100 μM) of PEL (**A**) or EPI (**B**) for 24, 48, and 72 h. Growth inhibition was measured using the neutral red analysis and is expressed as a percentage of DMSO-treated control cells (Ctrl).

The two secondary metabolites, PEL and EPI, isolated from the *Dactylospongia elegans* sponge, induced cell growth inhibition of 501Mel cells in a concentration- and time-dependent manner (Figure 2). In detail, PEL showed IC_50_ mean values of 12.51 ± 1.10, 4.17 ± 1.08, and 3.02 ± 1.06 μM after 24, 48, and 72 h of treatment, respectively (Figure 2A); EPI showed IC_50_ mean values of 7.88 ± 1.08, 5.71 ± 1.07, and 1.72 ± 1.10 μM after 24, 48, and 72 h of treatment, respectively (Figure 2B).

These data are similar to those obtained in a set of experiments on other skin cancer cells belonging to the squamous carcinoma A341 cell line, while significantly greater IC_50_ values were observed with the sponge metabolites when evaluated on the non-cancer skin cells HaCat (see Table S1).

In the following experiments, 501Mel cells were treated with 4 μM PEL or 5 μM EPI (concentrations near the relative IC_50_ values in the same cells) for 48 h.

### 2.2. PEL and EPI Induced Cell Cycle Arrest in G1 Phase

The analysis of the cell cycle profile of treated cells was examined by the evaluation of a marker of mitosis, Histone H3-pSer10, and a marker of the G1/S transition, Cdk2-pTyr15, to explore the mechanism underlying the inhibition of melanoma cell viability. In the late G2 phase and during mitosis up to the prophase, phosphorylation of H3 on Ser10 occurs up to the end of mitosis, when H3 is completely dephosphorylated [27]. Cdk2, on the other hand, is a master regulator of G1/S transition [28], which is triggered when Cdk2 is in the dephosphorylated active form, whereas it is prevented when Cdk2 is inactivated by phosphorylation on Tyr15.

**Figure 3:**
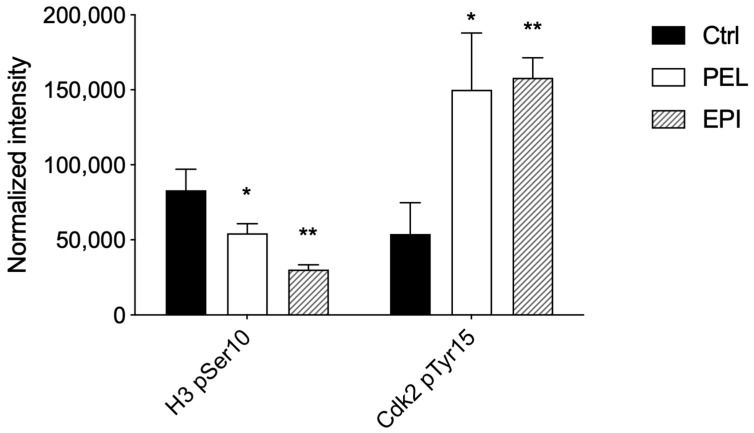
**Pelorol (PEL) 5–*epi*–ilimaquinone (EPI) induced a cell cycle arrest in the G1 phase.** Phosphorylation levels of Histone H3 at pSer10 and Cdk2 at pTyr15 were expressed as fluorescence units normalized on the corresponding cell amount (normalized intensity). Melanoma cells were treated with PEL 4 µM or EPI 5 µM for 48 h. Data are shown as means ± SD of three independent experiments, each performed in triplicate. Student *t*-test was performed; * *p* < 0.05 and ** *p* < 0.01 compared to the corresponding control (vehicle-treated cells, Ctrl).

After treatment with PEL at 4 μM, we showed that H3-pSer10 levels were significantly reduced (≅34%), and Cdk2-pTyr15 levels were significantly increased (≅178%) compared to control cells (Figure 3).

Similarly, in EPI-treated 501Mel cells, we observed a reduction in H3-pSer10 levels (≅63%) and an increase in Cdk2-pTyr15 levels (≅192%) compared to control cells (Figure 3).

Therefore, the significant reduction in the phosphorylation of H3 on Ser10 and the high phosphorylation levels of Cdk2 on Tyr15 in PEL- and EPI-treated melanoma cells suggested an arrest of melanoma cells in G1.

### 2.3. PEL and EPI Induced Apoptosis

The role played by the apoptotic process in the PEI and EPI-induced cytotoxicity was evaluated by measuring internucleosomal DNA fragmentation, an event that occurs during late phases of programmed cell death, and by analyzing the mRNA expression of the apoptotic genes BAX, BCL2, BIRC5, and MCL-1 and confirming their modulation through the evaluation of their upstream regulatory miRNAs, i.e., miR-214-3p, miR-16-5p, and miR-193a-3p, which have these genes as validated targets [29,30,31,32].

**Figure 4:**
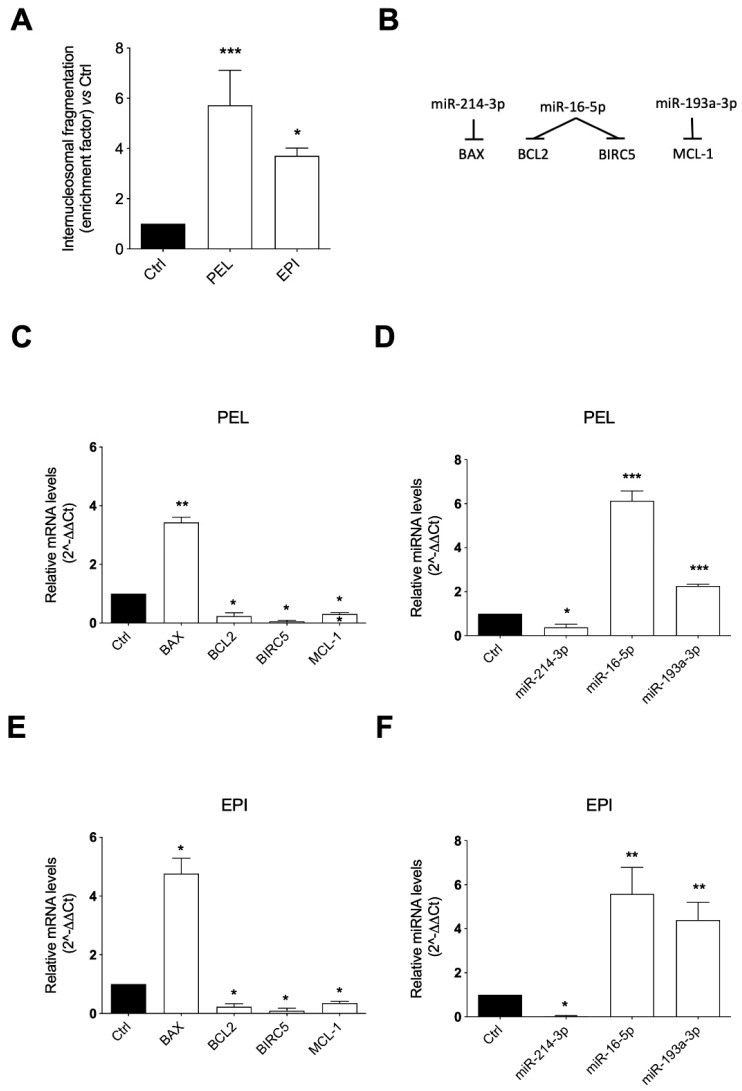
**Pelorol (PEL) and 5–*epi*–ilimaquinone (EPI) induced apoptosis in melanoma cells.** (**A**) Internucleosomal DNA fragmentation in 501Mel cells treated with 4 μM PEL or 5 μM EPI for 48 h, compared to DMSO-treated control cells. (**B**) Schematic representation of the miRNA-mRNA targeting. Expression levels of mRNAs (**C**,**E**) and miRNAs (**D**,**F**) involved in the apoptotic process in cells treated with 4 μM PEL (**C**,**D**) or 5 μM EPI (**E**,**F**) for 48 h, expressed as fold over control. Data are presented as means ± SD of three independent experiments, each performed in triplicate. Student *t*-test was performed. * *p* < 0.05, ** *p* < 0.01, *** *p* < 0.001, compared to the corresponding control.

The results showed that both PEL and EPI induced a significant accumulation of DNA histone fragments in the cytoplasmic fraction of the 48 h cell lysates compared to the control, slightly less prominent with EPI than PEL, indicating a pro-apoptotic effect of both compounds (Figure 4A).

The ability of PEL and EPI to induce apoptosis was proved by the analysis of the concordance in the expression levels of genes and miRNAs involved in the apoptosis process (Figure 4B). In detail, the 48 h cell exposure to PEL induced a robust increase in the pro-apoptotic BAX mRNA levels and a strong decrease in the anti-apoptotic BCL2, BIRC5, and MCL-1 mRNA levels (Figure 4C). The same trend was observed in melanoma cells treated with EPI, which significantly increased the mRNA expression of BAX and decreased the mRNA expression of BCL2, BIRC5, and MCL-1 (Figure 4E).

The levels of selected miRNAs able to post-transcriptionally regulate the mRNA expression of BAX, BCL2, BIRC5, and MCL1 were measured to examine the influence of the two sponge-derived compounds on this epigenetic control of gene expression. Concordantly with the gene modulation results, a significant reduction in miR-214-3p, targeting BAX, and a significant up-regulation of miR-16-5p, targeting BCL2 and BIRC5, and of miR-193a-3p, targeting MCL-1, were observed in PEL (Figure 4D) and EPI (Figure 4F)-treated melanoma cells.

## 3. Discussion

In recent years, the treatments of patients with cutaneous melanoma have significantly advanced with the development of immunotherapy and targeted therapy. However, additional therapeutic options still represent a clinical need, particularly for those patients who do not respond and/or relapse [10,11]. Growing evidence underline the presence of novel and interesting bioactive molecules in the blue environment, particularly sponge-derived molecules that appear to be promising future lead compounds in anticancer therapy.

**Figure 5:**
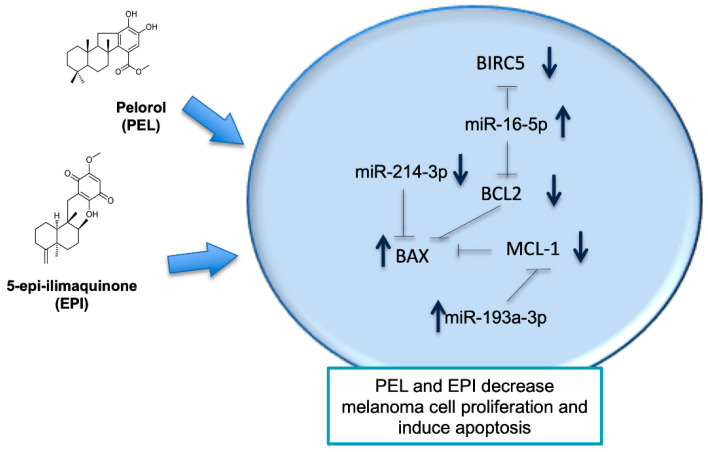
Schematic summary of the pathway implicated in the activity of Pelorol (PEL) and 5-*epi*-ilimaquinone (EPI) in melanoma cells (501Mel). The two secondary metabolites isolated from the marine sponges decreased melanoma cell viability and increased apoptosis controlling the expression of involved miRNAs and genes. See text for further details. Dashed lines indicate inhibition; the blue arrows indicate a decrease or an increase in gene or protein expression after treatment with PEL or EPI.

In the present study, we demonstrated that PEL and EPI, two terpenoidal derivatives present in marine sponges such as *Dactylospongia elegans*, decrease the viability of human cutaneous melanoma cells (501Mel) with suitable potency. We showed that PEL and EPI: (i) decreased melanoma cell viability, (ii) induced cell cycle arrest, and (iii) apoptosis by modulating genes and related miRNAs (see the schematic picture in Figure 5). These newly discovered effects of PEL and EPI as apoptosis-inducers and miRNA-modulators make them attractive molecules in anti-melanoma pharmacological research.

Herein, we showed that PEL and EPI exerted a significant decrease in cell viability in melanoma cells (501Mel) in a comparable manner to that observed by cisplatin [33], a conventional cytotoxic agent currently used for the treatment of melanoma [34].

Among the factors that can lead to limited or no response and/or resistance to drugs, the unserviceable apoptosis machinery and the control of alternative compensatory signaling, including miRNAs, play a crucial role [35].

Apoptosis is the cell death that occurs through a coordinated cell disintegration characterized by chromatin condensation, membrane blebbing, and nuclear fragmentation [36]. The intrinsic pathway of apoptosis is under the strict control of the actors of the Bcl-2 family, which comprises subfamilies with both pro- and anti-apoptotic roles. BAX is one of the main apoptosis effectors, which induces, after activation, the release of cytochrome c. Contrarily, BCL-2 and MCL-1 represent pro-survival and anti-apoptotic members of the Bcl-2 family [37]. Another important player in the apoptotic process is BIRC5 (survivin), a member of the inhibitor of apoptosis protein (IAP) family, involved in melanoma drug resistance [38].

A decrease in cell viability has been previously found for both compounds in different tumor cell models [20,21,26]. Potential underlying mechanisms included the ability to modulate, alone and in combination with conventional anticancer drugs, including cisplatin, the DNA damage stress response [26], and to induce the G1 arrest of the cell cycle and the up-regulation and nuclear translocation of the growth arrest and DNA damage-inducible gene 153 (CHOP/GADD153) [39]. Here, we report the ability of PEL and EPI to cause apoptosis in melanoma cells through the contemporaneous increase in pro-apoptotic BAX mRNA expression and decrease in the anti-apoptotic BCL-2, BIRC-5, and MCL1 mRNA expression. Therefore, the two sponge-derived terpenoids decreased cell viability by modulating the expression of genes involved in the apoptotic process. Though changes in the corresponding protein expression levels of BAX, BCL-2, BIRC-5, and MCL1 need to be verified, their modulation by PEL and EPI, together with other published data on the reduction in cell viability of PEL and EPI, coupled with anti-inflammatory activities [17,18,19], disclose the potential protective properties for these compounds against cancer, and their role as candidate compounds for further preclinical studies.

In an attempt to confirm the efficacy of EPI and PEL on apoptosis effectors, we explored the EPI and PEL effects on epigenetic regulators of gene expression and, in particular, on miRNAs, a class of naturally occurring, small non-coding RNA molecules (21–25 nucleotides), which function downregulating gene expression by translational repression or mRNA cleavage [40]. They are receiving growing attention as biomarkers as well as diagnostic and therapeutic targets because they regulate several biological processes and have been found to be dysregulated in human diseases, including cancer [41]. Indeed, miRNAs play a role in the control of tumorigenesis and drug resistance, and a single miRNA can concurrently bind to multiple transcripts affecting their expression. Particularly, the increased expression of oncogenic miRNAs and/or decreased expression of tumor-suppressive miRNAs lead to tumor cell proliferation and drug resistance [40]. Correction of altered miRNAs expression by their mimics or inhibitors has indeed been developed as a potential therapeutic approach [42,43].

BAX is a validated target of miR-214-3p [29], while BCL-2 and BIRC-5 are validated targets of miR-16-5p, and MCL1 of miR-193a-3p [29,30,31,32]. Therefore, we assessed EPI and PEL effects on the expression levels of these apoptosis-related miRNAs, expecting an opposite direction of changes compared to their target mRNAs. Indeed, we found a decrease in miR-214-3p and an increase in miR-193a-3p and miR-16-5p, in agreement with the corresponding modulation of their target mRNAs.

miR-214-3p has been demonstrated to function as both a tumor suppressor and an oncogene in various types of human cancer [44,45]. miR-214 has been shown to be highly expressed in human melanomas and to contribute to disease progression and metastases by targeting genes implicated in the migration, invasion, extravasation, and survival of melanoma cells [46]. Whether the inhibition of miR-214 expression by EPI and PEL translates, besides apoptosis induction, into additional anticancer effects and whether the pro-apoptotic effect is also mediated by other targets of miR-214 in addition to BAX [47] warrant further investigations. A role for miR-193a-3p as a tumor suppressor has been recently demonstrated in melanoma cells, in accordance with reduced plasma levels in melanoma patients [48]. Furthermore, a miRNA signature incorporating miR-16-5p has recently demonstrated a prognostic value in predicting brain metastasis in primary melanoma [49]. Further in vitro and in vivo investigations are needed to confirm the involvement of miR-214-3p, miR-16-5p, and miR-193a-3p in the anti-melanoma activities of PEL and EPI, using miRNA mimics and inhibitors. Collectively, our data indicate that PEL and EPI exert their cytotoxic activity against melanoma cells, promoting apoptotic signaling and inducing changes in miRNA expression and their downstream effectors. The capability of these two terpenoids to modulate non-coding RNAs, such as miRNAs, is in accordance with the relevant role of miRNA-based therapeutics in cancer [42,43] and represents a novel multifaceted mechanism of anticancer efficacy of the tested compounds.

## 4. Materials and Methods

### 4.1. Cell Line

501Mel human melanoma cells (from melanoma metastasis) were kindly provided by Dr. Poliseno (Oncogenomics Unit, Core Research Laboratory, Istituto Toscano Tumori c/o IFC-CNR, Pisa, Italy). 501Mel were cultured in RPMI 1640 medium (Euroclone, Milan, Italy) supplemented with 10% fetal bovine serum (FBS), 100 μg/mL streptomycin, and 100 U/mL penicillin (Euroclone, Milan, Italy) in a humidified atmosphere containing 5% CO_2_ at 37 °C.

### 4.2. Pelorol (PEL) and 5-epi-Ilimaquinone (EPI) Extraction and Purification

The extraction and purification of PEL and EPI from *Dactylospongia elegans* were performed as previously reported [21]. The sponge *Dactylospongia elegans* was collected on the island of Ambon (Indonesia) by SCUBA diving in December 2014. Interestingly, the presence of sponges of the Dactylospongia genus, including *Dactylospongia elegans* is also reported in the Red Sea, Saudi Arabia area [50,51]. Briefly, a solid residue (5.35 g) was obtained by concentrating under vacuum a methanol extract prepared from 130 g of freeze-dried sponge material. Then, the solid residue was dispersed in water (300 mL) and was successively fractionated against *n*-hexane, ethyl acetate, and *n*-butanol each of 1 L. Each fraction was separately evaporated under reduced pressure until getting solid residues of 2.71, 1.40, and 0.88 g, respectively. Afterward, the *n*-hexane fraction (2.71 g) was then chromatographed using silica gel 60 (250 g) as a stationary phase implementing a gradient elution procedure of *n*-hexane-ethyl acetate (100:0 to 0:100) with a 15% interval change and 500 mL each fraction affording 7 subfractions (V1–V7). Subfraction V4 (207 mg), obtained by *n*-hexane:EtOAc (1:1), was subjected to preparative HPLC for final purification and yielding pelorol (PEL, 3.9 g) and 5-*epi*-ilimaquinone (EPI, 42.3 mg).

### 4.3. Cell Viability Assay

PEL and EPI effect on cell viability was measured in melanoma cells in a concentration range of 0.1–100 μM for 24, 48, and 72 h. Cell number was determined using the Neutral Red Assay (Sigma-Aldrich, Milan, Italy). After 24 h of seeding melanoma cells (5 × 10^3^ cells/well) onto 96-well plates, cells were treated with PEL, EPI, or vehicle control (Ctrl). Secondary metabolites were dissolved in dimethyl sulfoxide (DMSO) and diluted in the treatment medium immediately before starting the experiment. In cell cultures, the final concentration of DMSO never exceeded 0.33%. During treatment incubation, only 1% FBS-added medium was used to avoid any interactions with serum proteins. After 24, 48, and 72 h, 10 μL of a neutral red solution (1% acetic acid and 50% ethanol) was added to each well, and the cells were incubated for 2 h at 37 °C. Optical density at 540 nm was measured using the Infinite^®^ M200 NanoQuant instrument (Tecan, Salzburg, Austria). The viability of cells treated with PEL or EPI was reported as a percentage of Ctrl (100% cell viability).

### 4.4. Cell Cycle

The phosphorylation levels of Histone H3 on pSer10, denoting a mitotic cell with condensed DNA, and of cyclin-dependent kinase 2 (Cdk2) on pTyr15, denoting a cell in the G1/S transition, were examined by using the quantitative immunocytochemistry Cell Cycle In-Cell ELISA kit (#ab140363, Abcam, Cambridge, U.K.) assay. Concisely, 10^4^ 501Mel were seeded onto 96-well plates and incubated with PEL or EPI at the indicated concentrations or their vehicle (Ctrl). After 48 h, cells were fixed with 4% paraformaldehyde. Then, cells were incubated with 0.02% sodium azide to reduce the background signal. The Cdk2-pTyr15 and Histone H3-pSer10 levels were measured by the incubation with specific primary antibodies and then secondary antibodies, which generate a signal through two spectrally distinct fluorogenic substrates. Each signal was normalized to the total cell amount of the corresponding well by using Janus Green stain (normalized intensity).

### 4.5. Internucleosomal DNA Fragmentation

To qualitatively and quantitatively determine the cytoplasmic levels of histone-associated DNA fragments (mono- and oligo-nucleosomes) as markers of the apoptotic process, the Cell Death Detection ELISA plus kit (#11774425001, Sigma-Aldrich, Milan, Italy) was used, as previously reported [12].

### 4.6. Gene Expression Analysis

Total RNA was extracted using the RNeasy Mini Kit and then reverse-transcribed using the QuantiTect Reverse Transcription kit (Qiagen, Valencia, CA, USA), according to the manufacturer’s instructions. Real-time PCR was performed using SsoFast Eva Green Supermix (Ref. 172–5201; Bio-Rad, Hercules, CA, USA). The sequences of forward and reverse primers were the following: BCL2: (F) TCCATGTCTTTGGACAACCA and (R) C TCCACCAGTGTTCCCATCT; BAX: (F) TCTGACGGCAACTTCAACTG and (R) TTGAGGAGTCTCACCCAACC; BIRC5: (F) ACCAGGTGAGAAGTGAGGGA and (R) AACAGTAGAGGAGCCAGGGA; MCL-1: (F) CCAAGAAAGCTGCATCGAACCAT and (R) CAGCACATTCCTGATGCCACCT; β-actin: (F) 5- AACTGGACGGTAGAAGGTGAC and (R) 5- GACTTCCTGTAACAACGCATC. The mRNA expression was determined using the 2^−^^ΔΔCt^ method, and β-actin was used as housekeeping.

### 4.7. microRNA Expression Analysis

The purification and extraction of total cellular miRNAs were performed using the miRNeasy Mini Kit (Qiagen, Hilden, Germany). The extracted miRNAs were retro-transcribed by the miScript Reverse Transcription Kit (Qiagen, Germany), and the corresponding cDNA was diluted 1:3 in water. The miScript SYBR-Green PCR kit (Qiagen, Germany) was used to carry out qPCR in triplicate. Signals were detected on the MiniOpticon CFX 48 real-time PCR Detection System (Bio-Rad, Hercules, CA, USA). MiScript Primer Assays specific for hsa-miR-214-3p (MIMAT0000271), hsa-miR-16-5p (MIMAT0000069), hsa-miR-193a-3p (MIMAT0000459) and hsa-SNORD6 were purchased from Qiagen. The miRNA expression was measured using the 2^−^^ΔΔCt^, and the SNORD6 gene was used as housekeeping.

### 4.8. Statistical Analysis

Data were presented as mean ± standard deviation (SD) of at least three independent experiments. All statistical procedures were performed by commercial software (GraphPad Prism, version 7.0 from GraphPad Software Inc., San Diego, CA, USA). The concentration of PEI and EPI able to inhibit cell viability by 50% (IC_50_) was used in each experiment. qPCR results were presented as box plots. The Student’s *t*-test was carried out to compare two groups. For a comparison of more than two groups, one-way ANOVA was used. A *p*-value < 0.05 was set as statistically significant.

## 5. Conclusions

In conclusion, our study is the first demonstration of an anti-melanoma activity of PEL and EPI by controlling apoptosis and related gene expression in melanoma cells (501Mel) and the first evidence of their in silico direct interaction with the α subunit of the PI3K protein. Our in vitro results provide a novel basic knowledge about marine-derived molecules able to simultaneously control cell viability and related miRNAs, thus representing promising lead compounds in the anti-melanoma drug research. Several methods to provide synthetic PEL have already been described [18,20,52], representing an important step to overcoming PEL’s low natural abundance and proceeding with pharmacological studies. Further studies are necessary to recapitulate and expand these results in in vivo models in monotherapy and combination protocols.

## Data Availability

The data presented in this study are available on request from the corresponding author.

## Supplementary Materials

The following supporting information can be downloaded at: https://www.mdpi.com/article/10.3390/md20070427/s1, Table S1: The half-maximal inhibitory concentration (IC_50_) of PEL and EPI after treatment of A431 and Hacat cells for 72 h.
