# Supplementary material for: Pro-Apoptotic Activity of the Marine Sponge Dactylospongia elegans Metabolites Pelorol and 5-epi-Ilimaquinone on Human 501Mel Melanoma Cells
Source: Mar Drugs. 2022 Jun 28;20(7):427. doi: 10.3390/md20070427 (PMC9317990; doi:10.3390/md20070427)
Supplement: Supplementary file 1 [file marinedrugs-20-00427-s001.zip › marinedrugs-1764849-supplementary.pdf]

Supporting Information

Table S1. The half-maximal inhibitory concentration (IC_{50}) of PEL and EPI after treatment of A431 and Hacat cells for 72 h.

	IC_{50} (μM) ± S.E.	
Compound	A431	HaCat
PEL	3.63 ± 1.08	50.04 ± 1.70
EPI	0.59 ± 1.29	53.92 ± 1.06
